# Sustainable palm weevil farming as nutrition supplementation at maternity waiting homes in Liberia

**DOI:** 10.1186/s12889-022-13706-8

**Published:** 2022-07-09

**Authors:** Christopher W. Reynolds, Madison Horton, Jacob Paarechuga Anankware, Joseph Perosky, HaEun Lee, Aloysius Nyanplu, Barsee Zogbaye, Alphonso Kofa, Jody R. Lori

**Affiliations:** 1 University of Michigan Medical School, University of Michigan, Ann Arbor, MI USA; 2 University of Michigan School of Nursing, Ann Arbor, MI USA; 3 Department of Horticulture and Crop Production, School of Agriculture and Technology, University of Energy and Natural Resources, Bono region, Ghana; 4 AnePare Farms, Sunyani, Bono region Ghana; 5 Spectrum Health, Grand Rapids, MI USA; 6 Center for Global Health Equity, University of Michigan, Ann Arbor, MI USA; 7 Bong County Health Team, Suakoko District, Bong County, Liberia

**Keywords:** Food insecurity, Edible insects, Maternity Waiting Homes, Nutrition, Global health

## Abstract

**Background:**

Food insecurity is a global health challenge exacerbated by COVID-19. In Liberia, two-thirds of pregnant women are anemic, one-third of children are stunted, and 70% of households experienced food insecurity due to COVID-19. Edible insects are a nutritious, environmentally responsible, and cost-effective dietary supplement used throughout sub-Saharan Africa. Rearing palm weevil insects at maternity waiting homes (MWHs)—residential dwellings near hospitals where pregnant women await childbirth and receive postpartum services—could serve as a nutritious supplement for expectant mothers in Liberia and provide an income generating activity for MWHs.

**Methods:**

Following a one-day training, sixteen participants established palm weevil rearing sites at four MWHs in Liberia. Pre- and post-knowledge scores were assessed immediately prior to and following training. Pre-and post-knowledge scores were analyzed using paired t-test. Participants tracked two palm weevil rearing cycles (four months), using harvest amounts, turnover, barriers to implementation, and income generated as metrics. The number of women attending MWHs was recorded throughout the study period (July-December 2020).

**Results:**

Sixteen participants from four MWHs completed the training and two rearing cycles (four months) successfully. All participants showed statistically significant increases in knowledge scores following the one-day workshop with a pre-test score of 2.31 and post-test score of 7.75 out of 10 (*p* < 0.001). Over the 6-month study, 217 women stayed in four MWHs. Larval production from the various rearing centers ranged from 120 to 721 larvae, with all four sites producing enough palm weevil to sustain MWH residents who desired to consume the insects. One site successfully commercialized its harvest to sell approximately 50% for a total of 2,000 LD (13 USD) in income. Three of the four sites continued edible insect production beyond the four-month study period.

**Conclusions:**

An edible insect project using palm weevil larva is one promising intervention as a nutrition supplement for expectant mothers at pre-established MWHs in rural Liberia. Edible insect rearing also has potential as an income generating activity for MWHs. Future studies should focus on addressing common barriers of remote implementation and metric tracking during the COVID-19 pandemic and reinforcing infrastructure to protect larvae rearing supplies.

**Supplementary Information:**

The online version contains supplementary material available at 10.1186/s12889-022-13706-8.

## Introduction

Food insecurity continues to be a global public health challenge. Current estimates show that nearly 700 million people are hungry, with most of these persons living in low and middle-income countries (LMICs) [1]. Recognizing this problem, the United Nations called for “Zero Hunger” by the year 2030 in their Sustainable Development Goals [2]. Despite this aspiration, food insecurity has worsened due to climate change affecting food production through increasing temperatures, changing precipitation patterns, and extreme events [3]. Since 2020, the COVID-19 pandemic has caused additional disruptions to supply chain management, upset the economic stability of farmers, and threatened both crop harvests and distribution of nutritious food [4].

Particularly in LMICs, food insecurity is compounded by preexisting poverty, causing disproportionately widespread undernourishment compared to other higher-income countries [5]. Women and children are especially at risk; it is estimated that undernutrition is the underlying cause of over one-third of child deaths and 10% of the total worldwide burden of disease [6]. Providing adequate nutrition, including balanced protein and iron intake, to pregnant women has been shown to reduce the incidence of small-for-gestational age births, stillbirths, and low birthweight newborns [5, 7].

Food insecurity during pregnancy is a significant problem in West Africa, where every fifth household is considered food insecure, mainly affecting poor rural households. Liberia has suffered historically from colonial exploitation, and more recently from a 14-year civil war which destroyed infrastructure and basic social services, contributing to widespread poverty and food insecurity [8]. The 2014–16 Ebola epidemic, which resulted in nearly 5,000 deaths, further exacerbated food insecurity due to traveling and trade restrictions, raised crop prices, and stunted income from direct morbidity and mortality caused by the virus [9, 10]. The current COVID-19 pandemic and these restrictions caused by the virus have worsened Liberia’s food insecurity. Travel and trade restrictions have increased food prices of domestic supply, decreased earning potential for harvests, and increased access difficulties for NGOs and governmental programs to deliver nutritious food to at-risk families [11]. Nearly 30% of Liberian households expressed need for assistance related to livelihood support from high food insecurity due to COVID-19 [11]. In Bong County, 79% of households reported a lack of adequate food security even before the COVID-19 pandemic, with 51% reporting moderate to severe chronic food insecurity [12].

These barriers to food security have significant health effects. In Liberia, chronic malnutrition is estimated to affect 32% of the population and is the number one factor driving death and disability [13]. Pregnant Liberian women and their children are especially at risk, as nearly two-thirds of pregnant women are anemic and one-third of children under age five suffer from stunting [14]. Nationally, 8% of Liberian women of childbearing age are chronically undernourished, and forty percent of Liberian girls begin childbearing before age 18, putting them at higher risk for malnutrition [15]. The consequences of maternal undernutrition in Liberia can be attributed to 12% of child deaths, stunting of growth, and a determinant of obesity and non-communicable diseases later in life [16]. One study examining food insecurity among pregnant Liberian women demonstrated increased vulnerability with positive correlations between malnutrition, HIV, and intimate partner violence [17].

Despite the need to address food insecurity, sustainable food programs have been difficult to implement in Liberia amidst shifting national priorities and instability from infectious disease pandemics and post-conflict transition following civil war. While strategies such as crop modification and various income-generating activities (IGAs) have been proposed as potential solutions to food insecurity, very little literature is specific to sub-Saharan Africa, and none explicitly addresses the impact of such interventions on the planet or considers long-term sustainability [18]. One noteworthy approach is the consumption of nutritious indigenous insects as a safe dietary supplement, which can also serve as an IGA if insects are commercially harvested [19–21]. Studies have found that insects provide four times the amount of iron and ten times the amount of protein as beef, while also directly addressing the climate crisis [22, 23] (Additional file 1: Appendix 1) Compared to animals, the cultivation of edible insects has minimal impact environmentally and are products of typical waste, as adult palm weevils are harvested from dead palm trees usually felled to pave way for new plantings [24]. Additionally, palm weevil farming promises economic sustainability by using alternative feed substrates including sugar cane, pawpaw, brewery waste, and coconut husk waste that is typically discarded.

An acceptability study conducted by our team with over 200 participants across Liberia demonstrated that insect consumption was viewed as an acceptable and common form of dietary supplementation for pregnant women [25]. Focus group members highlighted the year-round availability and high marketability of the palm weevil larvae, which makes them an ideal species to utilize as a complementary source of nutrition for pregnant women in Liberia [24]. As a good source of dietary supplementation and feasible method of IGA according to relevant stakeholders, palm weevil farming could be a promising method to address undernutrition among pregnant women in Liberia.

This study implemented edible insect projects at four maternity waiting homes (MWHs) in Liberia. MWHs are residential dwellings located near health facilities where women in the late stages of pregnancy stay to await childbirth and receive immediate postpartum services [25]. A USAID funded project initially began with a study of six MWHs in 2010 to examine MWHs as an intervention to reduce maternal and neonatal deaths by increasing access to facility-based delivery [25]. A follow up country-wide assessment of MWHs throughout Liberia in 2019 led to a scale-up to 114 MWHs. However, food insecurity was identified as a major barrier to women using these facilitates [26]. Therefore, MWHs served as an ideal site to pilot this project, as they are staffed by certified healthcare professionals who, along with community members, have identified nutritious food access among pregnant women as a problem that can be addressed by this intervention.

## Methods

### Collaborative design

This intervention was a unique public–private partnership between The University of Michigan, AnePare Farms, and the Bong County Health Team in Liberia. AnePare Farms provided expert consultation and supervision of training of palm weevil farming based on methodologies proven successful in Ghana. AnePare Farms developed an innovative approach to rearing edible insects using simple tools, including a bucket and mesh screen, packaged as a rearing kit. The ‘insect farmer’ needs three minutes every two days to attend to the rearing kit until insect maturity in 21 to 28 days, at which time matured larvae were ready for harvesting. Rearing the palm weevil from an adult to the consumable larval stage takes only 21 to 28 days depending on the environmental conditions, the quality of the feed, and the presence or absence of parasitoids. A complete lifecycle for continuous rearing takes 60 days. The Bong County Health Team is a branch of the central Ministry of Health, whose members coordinated and conducted trainings at the rural MWHs, purchased start-up supplies, and provided ongoing evaluation and data collection for the project duration.

### Intervention training

In January of 2020, two members of the Bong County Health Team traveled to Ghana to participate in training at AnePare Farms. All participants provided informed consent before program enrollment. They were trained on how to start and maintain a palm weevil farm. These trainees served as lead trainers who returned to Liberia to conduct one half-day workshop and trained a total of 16 participants at four MWH sites: Naama, Phebe, Fenutoli, and Janyea.

Participants were trained to start-up, maintain, and process edible insects as a sustainable food source for pregnant women at a MWH and were provided with starter materials for the construction of larvae storage facilities. Participants were also trained to process the palm weevil larvae into food powder, cubes, protein bars, pies, cakes and as a weaning feed for infants. The estimated startup costs for supplies and logistics to begin farming was 600USD per site. Participants were asked to record daily logs on larvae production worksheets. The goals of this intervention were to: i) develop local knowledge for sustainably implementing an edible insects training program; ii) document successful larvae production from participants’ rearing kits after two complete cycles; and iii) evaluate whether income generation through insect farming would generate a source of revenue at rural MWHs.

### Data collection and analysis

Participant understanding of palm weevil farming, edible insect nutrition facts, maintenance, and processing was assessed with pre- and post-training knowledge assessment surveys immediately prior to and after the training. Following knowledge assessment surveys, participants were supplied with mixed methods project evaluation tracking sheets to document edible insect harvests, turnover cycles, barriers faced during project implementation, and potential income generated through palm weevil production.

Data on MWH attendance over the study period (July-December 2020) was captured by The Bong County Health Team, which was collected from midwives and trained birth attendants staffing MWHs. Pre- and post-training knowledge assessment surveys, evaluation tracking data, and MWH census data were manually entered and cleaned in Microsoft Excel. SPSS was used for descriptive statistics. Paired t-test was used to examine the differences in the pre-and post-knowledge assessment tests. Qualitative data were grouped into repetitive themes, and varied quotes were selected to highlight relevant aspects of project implementation.

## Results

### Population

**Fig. 1:**
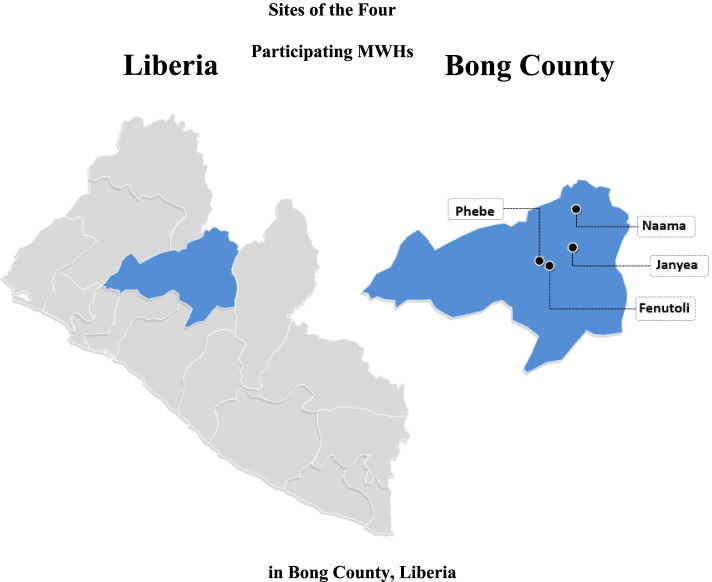
Sites of the Four Participating MWHs in Bong County, Liberia. This figure demonstrates Bong County and the locations of the four participating Maternity Waiting Homes throughout the research study period. The left map displays Liberia and its 15 counties, with Bong County highlighted in blue. The right map demonstrates the location of each participating MWH, which is indicated with a black dot and its name

**Fig. 2:**
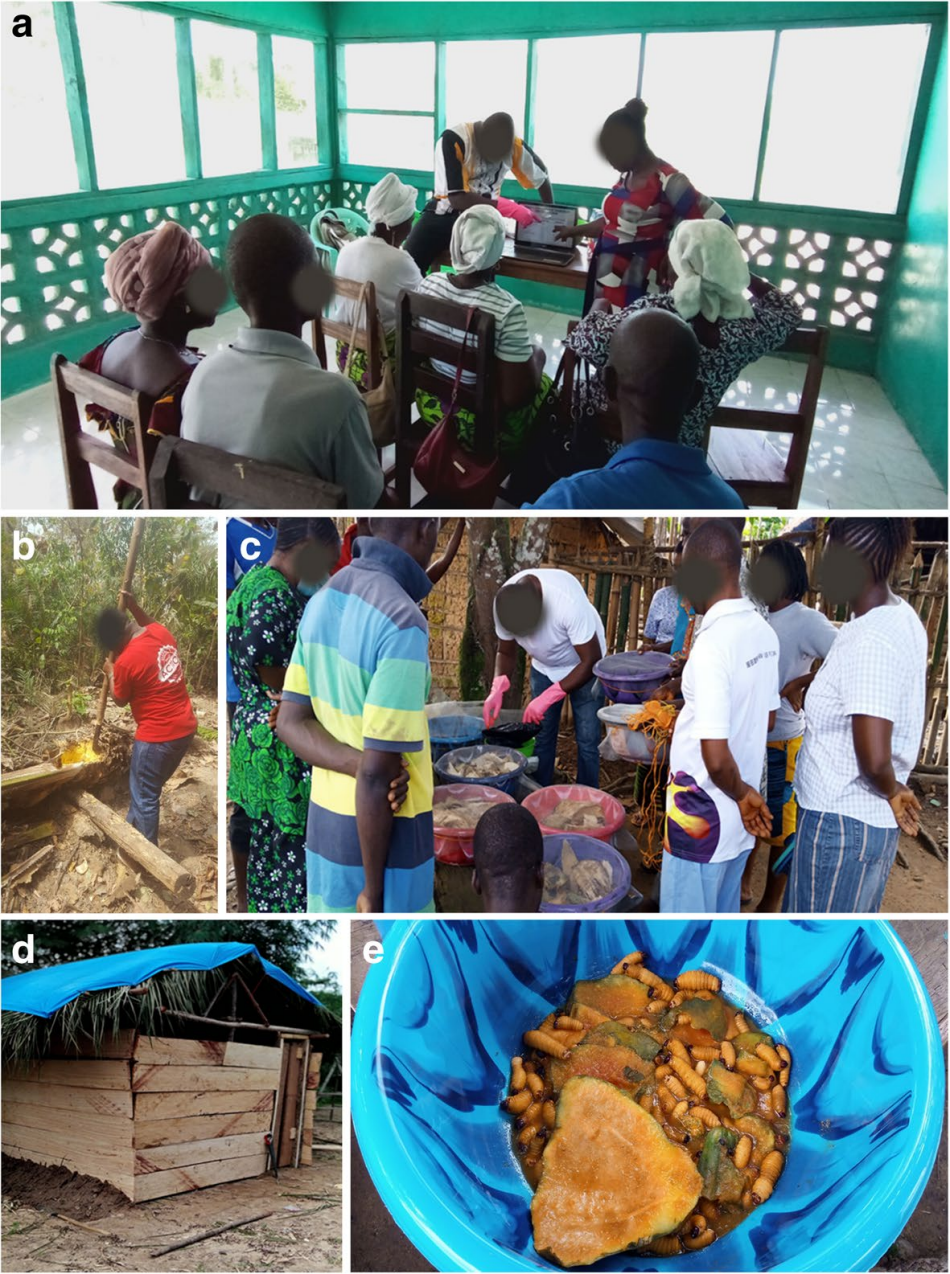
**a**-**e** Palm Weevil Project Training and Implementation. **a** Palm weevil training with The Bong County Health Team at one of the four participating MWHs; **b** Harvesting palm yolk for larvae feed; **c** Wooden rearing house used to store palm weevil inoculation bins; **d** Training community members in palm weevil rearing; **e** Palm weevil harvest at Fenutoli

Four MWHs were selected based on their willingness, staff availability, and close working relationship with The Bong County Health Team. Training was successfully conducted for 16 participants across the four MWHs: Naama, Fenutoli, Janyea and Phebe (Fig. 1). Participants were provided with palm larvae rearing kits, starter culture of adult palm weevil, larvae and cocoons, and a wooden structure for their insect colonies. Participants consisted of community health workers, public health consultants, pregnant women, opinion leaders and local leaders. Larvae farms were established at participating MWHs according to training guidelines, constructed as a wooden structure with thatched and tarpaulin roof. Wooden stands served as the shelves to hold bins used for larvae inoculation (Fig. 2a-e).

Over the 6-month study period (July-December 2020), 751 women gave birth across the four hospitals associated with MHWs, with a total of 217 women staying in MWHs (Table 1).

**Table 1:** Average number of deliveries and number of women staying at MWH (July-December 2020)

	**Average Number of Deliveries/month (N)**	**Average number of women staying at MWH/month (N)**
Total	125.2	26.17
Facility		
Phebe	49.33	5.83
Naama	29.17	11.50
Janyea	16.67	5.67
Fenutoli	30.00	13.17

### Training and implementation

Among 16 participants who underwent the training, all participated in pre- and post-training knowledge score evaluations, and implementation monitoring over the course of 2 cycles of larvae production during the project implementation phase. There was a statistically significant difference in eight out of nine knowledge areas including the nutritional information, start- up, maintenance, and processing of palm weevil before and after the training (Tables 2 and 3).

**Table 2:** Pre- and post- training knowledge test descriptive statistics

	**Pre-test (*N* = 16) n (%)**	**Post-test (*N* = 16) n (%)**	***p*-value**
**Overall score, mean (SE)**	**2.31 (0.45)**	**7.75 (0.39)**	
There is more protein in palm weevils than in chicken	6 (37.50)	16 (100.00)	< .01**
There is more protein in palm weevils than in beef	8 (50.00)	16 (100.00)	< .01**
There is more protein in palm weevils than in fish	5 (31.25)	16 (100.00)	< .001***
Insect farming is more sustainable than livestock farming	5 (31.25)	15 (93.75)	< .01**
Palm weevil larvae matures in which stage?	0 (0)	7 (43.75)	< .01**
Palm weevils have males and females	5 (31.25)	16 (100.00)	< .001***
How many eggs does a palm weevil lay after mating?	0 (0)	5 (31.25)	.02*
How long does it take for palm weevil eggs to hatch?	0 (0)	7 (43.75)	< .001 ***
You must feed palm weevil larvae every day	7(43.75)	13 (81.25)	.05
Which metamorphic (developmental) stage is consumed?	0 (0)	13 (81.25)	< .001***

^*^p < 0.05 ** *p* < 0.01 ***p < 0.001

**Table 3:** Pre-and post-training knowledge test paired samples statistics

	**Mean**	**N**	**Std. Deviation**	**Std. Error Mean**	**Significance**
Pre-test	2.31	16	1.81	0.45	< .001***
Post-test	7.75	16	1.57	0.39	

^*^p < 0.05 ** *p* < 0.01 ***p < 0.001

### Production metrics

All four sites successfully produced larvae of varying quantities from their rearing kits for two or more cycles (four months). The larval production from the various rearing centers was relatively low, ranging from 120 to 721 larvae harvested within the two production cycles (Table 4). Naama produced the most larvae at harvest, almost four times the second most productive MWH (Phebe).

**Table 4:** Larvae produced and consumed at the various rearing centers

Site	Larvae harvested	Larvae diverted	Larval mortality	Percentage of harvested larvae consumed	Total pieces consumed
Naama	721	300	80	100%	721
Phebe	200	107	48	50%	100
Janyea	187	102	21	7%	13
Fenutoli	120	84	17	62%	75

Larvae harvested: larvae produced after the 21–28 day period

All four sites reported using exclusively palm yolk as the larvae feed substrate. When participants were asked about the most preferred way to eat the harvested palm weevil larvae, 50% indicated the larvae were best eaten when grilled while 25% preferred to eat fried and 25% favored boiled larvae. Other consumption methods included roasting and using larvae as a condiment. Each site reported similar by-products from larvae production, including manure for future farming (100%), condiments (50%), firewood (25%), and protein for soups (25%).

Larvae consumption among residents varied across each site. Naama recorded the most significant acceptability and consumption measures among the four locations, with all insects produced also being consumed over the study period. Despite having the largest adult larvae production number at 721, its consumption percentages resulted in no commercialization of product for income generation beyond MWH residents. At Phebe, residents consumed about 50% of larvae for a total of approximately 100 pieces consumed and the remaining 100 commercialized for profit. Janyea had less consumption, with less than 10% of produced larvae being eaten. Participants reported differences across each site in how the edible insects were promoted to residents. At Phebe and Naama, there was strong participation from MWH staff and health care workers for promoting the project. They advertised the edible insect options as a nutritious supplement to residents upon their entry and throughout their stay at the MWHs, specifically by referencing the increased protein and calcium benefits of palm weevil consumption during pregnancy that was learned during their training. These staff incorporated rearing of the insects into their daily workflow. Janyea reported less interested staff and difficulty with supervision given distance to the rearing centers. Across the four sites, frequency of consumption ranged from only one time to a maximum of 10 pieces per day, depending on preferences of each resident. MWH residents who rejected consuming edible insects even when they were available did so because some indigenous tribes do not consume insects. Other residents reported that the palm weevils were “like a worm,” and some simply did not find them appetizing.

Only one MWH produced enough larvae for sale as well as consumption (Phebe), where approximately 50% of the production was consumed by pregnant women staying at the MWH and 50% sold for a profit. Larvae were sold as packs of five or six insects for 100LD (0.65USD). Phebe generated 2,000 Liberian dollars over 2 months (approximately 13 USD) from its palm weevil profits. Participants attributed this success to the investment of local traditional birth attendants (TBAs) who showed high levels of commitment to larvae production and harvesting. Two sites produced mainly for consumption by MWH patients (Naama and Fenutoli). At Fenutoli and Naama, all palm weevils raised were either consumed by pregnant women staying at the MWHs or diverted to continue with additional rearing cycles. The final site, Janyea, presented challenges as the larvae drowned after the second cycle due to structural instability of the rearing houses. Participants attributed the low insect consumption at Janyea to this barrier in adult larvae production.

Though 75% of sites were able to continue farming beyond two cycles, participants cited challenges to implementation and sustainability. The most common reasons included limited feed for the larvae (100%) and lack of staff motivation (25%). All four sites reported that the farms were “easy to maintain” when appropriate amounts of feed were available, including for harvesting process and waste disposal. Two participants trained others in the palm weevil farming technique, for a total of three new trainees.

## Discussion

Food insecurity and undernutrition in LMICs including Liberia continues to be a serious public health challenge, contributing to preventable maternal and neonatal morbidity and mortality. Innovative and sustainable interventions are needed to combat this under-resourced global need. An edible insect project, using palm weevil larva as complementary diet for expectant mothers at pre-established MWHs in rural Liberia, is a promising intervention to provide supplemental nutrition and broaden sources of protein. This pilot study shows the intervention was successfully implemented across four MWH sites, requiring minimal training and resources. After one training session, participants showed considerable knowledge gains, and were able to successfully implement and sustain low-cost and low-resource palm weevil farming. Participants reported that maintaining the larvae farms and tracking the production were easy to do, and that production was effective enough to provide palm weevil as food to all desiring expectant mothers at the MWHs.

In addition to serving as a nutritious food supplement for pregnant women awaiting delivery, this intervention served as a modest income generating activity for one project site in a short amount of time. The site produced enough product to generate income from larva profits, earning 13 USD over the study period which was used to purchase additional foods and other needed resources to deliver quality care to pregnant women. Though Phebe grossed only 13 USD over the two-month study period, this figure showed positive economic profit at one site following a one-time initial startup cost. With approximately 50% of households in Bong County living in extreme poverty and making less than 1.25USD per day [27], positive income generating activities can be a helpful additional benefit of this program for MWHs in this region. Furthermore, by-products of the farming provided additional resources, including crop manure and firewood. This finding is significant, as it relates to other economic models for sustainable revenues for the community.

There were discrepancies across the four sites in terms of production and consumption. Naama produced and consumed the most edible insects, likely given the large interest from MWH residents at this site. This differed from Phebe, who despite harvesting less than one-third that of Naama with only half as many residents, was also able to commercialize its extra product. We hypothesize that this difference may be due to variance in interest from residents and priorities for how to allocate larvae, as sites were given autonomy to commercialize their product. Despite the successes of these sites, Janyea conversely only reported 13 palm weevils being consumed. We hypothesize that this discrepancy may be due both to decreased interest and limited availability, as Janyea had less output comparatively, and suffered a significant loss of product due to flooding in the rearing houses which caused many of the edible product to drown. Rainwater drowning (Janyea) and lack of feed for continuous palm weevil larval production were factors that might have led to a reduction in output. Cocooning and adult hatching was low on average across all rearing centers compared to potential expected yield of 800 insects per site. This is the first study in Liberia to assess palm weevil farming, so there is limited data on true expected yields. Discrepancies may be due to parasitoids and predation, particularly at Fenutoli and Phebe. Parasitoids and predation are two factors in edible insect farming which present potential explanations for lower yield, as they disrupt the environmental conditions necessary for proper growth and reproduction and eliminate the number of total larvae produced. Natural selection may have also played a role in discrepancies among rearing sites. Although the colony started with artificial selection through the control and manipulation of feed (palm yoke and sugar) without environmental manipulation, natural selection produced offspring with unique and heritable fitness within the population to increase the fitness of the individuals in the subsequent generations. In nature, complex community interactions drive natural selection, which could be underpinned by the various environmental conditions across rearing centers. This process selects organisms which are most suited to survive in a given environment, while killing off those who do not possess such traits, making those with “fit” traits more likely to reproduce and occupy a population. For example, if a bacteria or fungi harmful to palm weevils had infiltrated the rearing center, this would cause a significant decrease in production outcome for the infected farm. Such a process or combination of factors could explain why Fenutoli and Phebe had differences in production amount, despite their close geographic proximity. Though this study was relatively short with two rearing cycles, future outcomes of long-term production should also consider discrepancies due to natural selection to increase the fitness of palm weevils in the subsequent generations.

Despite successes, there were barriers to project implementation and sustainability. All participants reported acquiring feed for the larvae as an additional barrier. Though all sites reported sufficient access to source palm yolk to continue with rearing, feed was one of the only materials that had to be continually supplied or bought by sites throughout the rearing period. Once the intervention is scaled to other MWHs, communicating during the training session of the need to replenish feeding supplies, establishing supply chains to source palm yolk and cassava feeds, and assigning specific project participants to be responsible for acquiring feed with ample time to not upset rearing cycles will be important to successful implementation. Second, Phebe was the only site able to commercialize and sell its extra larvae for profit. Participants attributed this success to invested staff committed to project success, emphasizing the need for recruiting project managers and TBAs who believe in the importance of the intervention and have the capacity to oversee the implementation. Less productive sites confirmed this finding, identifying lack of participants’ motivation as a barrier. This finding emphasizes the importance of recruiting enthusiastic leaders, as well as educating and empowering community members for engagement and project success. TBAs are a unique population with a special combination of community knowledge and trust alongside health care providers, who could serve as effective ambassadors to this program for MWH residents. As these interventions expand, it will be important to equip and empower TBAs and other MWH health care workers with the tools to successfully implement palm weevil rearing projects. Similarly, though participants were not expected to recruit or train others to assist with the project, two participants trained a total of three new trainees. This shows potential for a future train-the-trainer model as the intervention is scaled to a larger population. Infrastructure problems arose at the Janyea site, whose farm flooded following two cycles (four months). To avoid similar issues during Liberia’s rainy season, more sustainable rearing houses should be considered, such as metal roofs and concrete foundations.

A main challenge to program implementation was the difficulty with support and supervision from higher level trainers at the MWHs, as COVID-19 disrupted the ability to support early learners in a more hands-on approach. Phebe, one of the more successful sites, was located near the Bong County Health Team office, where lead trainers were more consistently available for consultation and support. Given this observation, providing close supervision and support to new trainees will be of crucial importance as the intervention is scaled up to other MWHs across Liberia. There are also opportunities for using more creative approaches to training, monitoring, and evaluating staff and program implementation, including tele-consults and use of mobile apps to track harvests, as in-person activities may continue to be limited. Additionally, the COVID-19 pandemic has exacerbated food insecurity for many Liberians, including MWH staff. Nearly 70% of participants from one study in Liberia reported new-onset food insecurity from the pandemic [28]. Guaranteeing that staff feel supported not only in rearing edible insects for MWH residents, but also their own food access, is paramount. These barriers affirmed that, education, awareness, and capacity-building paired with community support are essential to sustainability in an edible insect rearing project.

Though an effective solution for combating malnutrition given their high protein, lipid, vitamin, mineral, and fiber content, edible insects are a relatively new public health solution for combating undernutrition [29]. While emerging research has demonstrated the health benefits of edible insects, including antimicrobial, nutritional, and therapeutic properties [29], few studies have evaluated implementation of an edible insects programs among populations vulnerable to malnutrition. Most research advocating for edible insect consumption are observational studies on traditional practices [30], including in Madagascar [31], the Democratic Republic of Congo [32], and Thailand [33], or acceptability studies in Western countries without traditions of edible insects [34]. Acceptability studies in Liberia and Ghana have shown important stakeholders, including caregivers, patients, community leaders, and healthcare professionals, to have favorable opinions towards palm weevil larvae farming as a source of nutrition [19, 24, 35]. These studies confirmed our findings that palm weevil farming is low cost, sustainable with proper support, and acceptable to participants as a source of nutrition. A further benefit of our study demonstrates that this accepted intervention can be implemented into an existing health system. One observational study in South Africa demonstrated edible insect trading as a popular source of income which helped to improve rural livelihoods [36], exhibiting even more robust market value than our study for edible insects. Generally, our study adds to the growing body of literature on edible insect consumption to combat malnutrition. It is one of the only to implement and measure edible insects farming and consumption as a novel intervention, and the first to do so among pregnant women. This research is also the first to measure income-generating potential of palm weevil farming as a secondary benefit for health centers. This study could inform future efforts for scale-up to country-wide MWHs and supplementing nutritious food options in an environmentally sustainable way.

### Limitations

This study has several limitations. First, implementing this program with the onset of the COVID-19 pandemic presented significant challenges, including travel restrictions which prevented The Bong County Health Team from close monitoring of progress at each MWH. For future interventions, such close supervision which was vital to the project outcomes may be more difficult with increased sites. Scale-up should prioritize effective strategies for frequent check-ins in the context of COVID-19 while not overburdening supervisors. Second, the rearing houses were made of wood and were susceptible to unforeseeable events such as the flooding. Therefore, more durable farming supplies and material should be considered for future studies. Finally, data collection using paper tools was less convenient for researchers and participants and made follow-up more difficult among COVID-19 travel regulations. Such limitations could be mitigated with digitalized survey tools such as mobile apps.

## Conclusion

Palm weevil rearing at MWHs in Liberia can be a feasible and sustainable intervention to supplement nutrition among pregnant women, with potential for modest IGA. An edible insect project designed for pregnant women addresses many of the Sustainable Development Goals, mainly 2) Zero Hunger, 3) Good Health and Wellbeing, and 12) Responsible Consumption and Production [2]. This project successfully trained local participants for start-up, maintenance, and processing of edible insects, documented successful larvae production for two complete cycles, and evaluated income generation potential of insect farming at MWHs. Despite identified barriers across the four MWHs, each site successfully produced enough larvae for pregnant women, and one site successfully sold 50% of their product. The palm weevil farming demonstrates exciting potential for country-wide scale-up. Future projects should consider monitoring clinical outcomes related to pregnant women who consume edible insects compared with those who do not, and how to construct cost-effective but simultaneously durable insect rearing houses. Research at a country-wide level could provide data to further develop best practices and investigate the nutritional benefit of palm weevil consumption for pregnant women and children beyond those utilizing the MWHs.

## Supplementary Information

**Additional file 1: Appendix 1.** Comparison of Sources of Protein and Iron.

## Data Availability

The datasets used and/or analyzed during the current study are available from the corresponding author on reasonable request.

## Abbreviations

LMIC: Low- and middle-income countries
IGA: Income generating activities
MWH: Maternity Waiting Home
USD: United States Dollar
