# Supplementary material for: Sustainable palm weevil farming as nutrition supplementation at maternity waiting homes in Liberia
Source: BMC Public Health. 2022 Jul 9;22:1313. doi: 10.1186/s12889-022-13706-8 (PMC9270802; doi:10.1186/s12889-022-13706-8)
Supplement: Supplementary file 1 — Additional file 1: Appendix 1. Comparison of Sources of Protein and Iron. [file 12889_2022_13706_MOESM1_ESM.docx]

Appendix 1: Comparison of Sources of Protein and Iron

| Meat | Protein Content (g/100 g) | Iron Content (mg/100 g) |
| --- | --- | --- |
| Palm weevil Rhychophorus | 66.3 | 30.8 |
| Goat (Chevon) | 27.1 | 3.8 |
| Cow (Beef) | 27.4 | 3.4 |
| Fowl (Chicken) | 24.0 | 1.7 |
| Sheep/lamb (Mutton) | 25.6 | 1.6 |
| Fish | 23.3 | 0.9 |
| Duck | 11.5 | 2.7 |
